# NextGen Public Health Surveillance and the Internet of Things (IoT)

**DOI:** 10.3389/fpubh.2021.756675

**Published:** 2021-12-03

**Authors:** Kirti Sundar Sahu, Shannon E. Majowicz, Joel A. Dubin, Plinio Pelegrini Morita

**Affiliations:** ^1^School of Public Health Sciences, University of Waterloo, Waterloo, ON, Canada; ^2^Department of Statistics and Actuarial Science, University of Waterloo, Waterloo, ON, Canada; ^3^Institute of Health Policy, Management, and Evaluation, University of Toronto, Toronto, ON, Canada; ^4^Department of Systems Design Engineering, University of Waterloo, Waterloo, ON, Canada; ^5^Ehealth Innovation, Techna Institute, University Health Network, Toronto, ON, Canada; ^6^Research Institute for Aging, University of Waterloo, Waterloo, ON, Canada

**Keywords:** real-time data, rapid surveillance, data source, big data, innovation

## Abstract

Recent advances in technology have led to the rise of new-age data sources (e.g., Internet of Things (IoT), wearables, social media, and mobile health). IoT is becoming ubiquitous, and data generation is accelerating globally. Other health research domains have used IoT as a data source, but its potential has not been thoroughly explored and utilized systematically in public health surveillance. This article summarizes the existing literature on the use of IoT as a data source for surveillance. It presents the shortcomings of current data sources and how NextGen data sources, including the large-scale applications of IoT, can meet the needs of surveillance. The opportunities and challenges of using these modern data sources in public health surveillance are also explored. These IoT data ecosystems are being generated with minimal effort by the device users and benefit from high granularity, objectivity, and validity. Advances in computing are now bringing IoT-based surveillance into the realm of possibility. The potential advantages of IoT data include high-frequency, high volume, zero effort data collection methods, with a potential to have syndromic surveillance. In contrast, the critical challenges to mainstream this data source within surveillance systems are the huge volume and variety of data, fusing data from multiple devices to produce a unified result, and the lack of multidisciplinary professionals to understand the domain and analyze the domain data accordingly.

## Introduction

The function of public health systems is to understand and respond to health trends affecting populations (1). This is achieved through public health surveillance, that is, the ongoing collection and analysis of population health indicators. Traditional surveillance data collection can be cumbersome, expensive, and slow, often relying on paper-based and digitally extracted data sources. Social media and crowdsourcing are data sources that can be leveraged for surveillance data (2, 3). Sources like Twitter, Facebook, Google, and Reddit have been successfully used to explore behavior and health outcomes (4–6). These are now being accepted as potential data sources across several health domains (7, 8).

Another promising data source is the increasing number of devices (e.g., smart home monitors, wearables) and the technology to interconnect them. Internet of Things (IoT) technologies have become mainstream within communities and individual households (9). Wearables and sensors can track personalized parameters of healthy living, including sleep, physical activity, and sedentary behavior (10, 11). These devices can provide insights into population health, disease management, and active assisted living services (12, 13). IoT data has several advantages over traditional surveillance data: high volume and frequency of data collection, data triangulation, real-time availability, and minimal acquisition effort.

Existing literature discusses the potential use of the IoT data sources for different purposes within multiple domains including healthcare. Among healthcare domain, area specific application can be seen for pediatric, geriatrics, chronic disease supervision, private health, and fitness management (14, 15), but no single study exists to put together the views to utilize the IoT data with specific emphasis on public health surveillance.

This article summarizes the existing literature on the use of IoT as a data source for surveillance. We discuss the shortcomings of current data sources and how IoT can meet the needs of surveillance. Challenges facing the large-scale application of IoT data to surveillance are also explored.

## Public Health Surveillance and Challenges With Existing Data Sources

**Figure 1:**
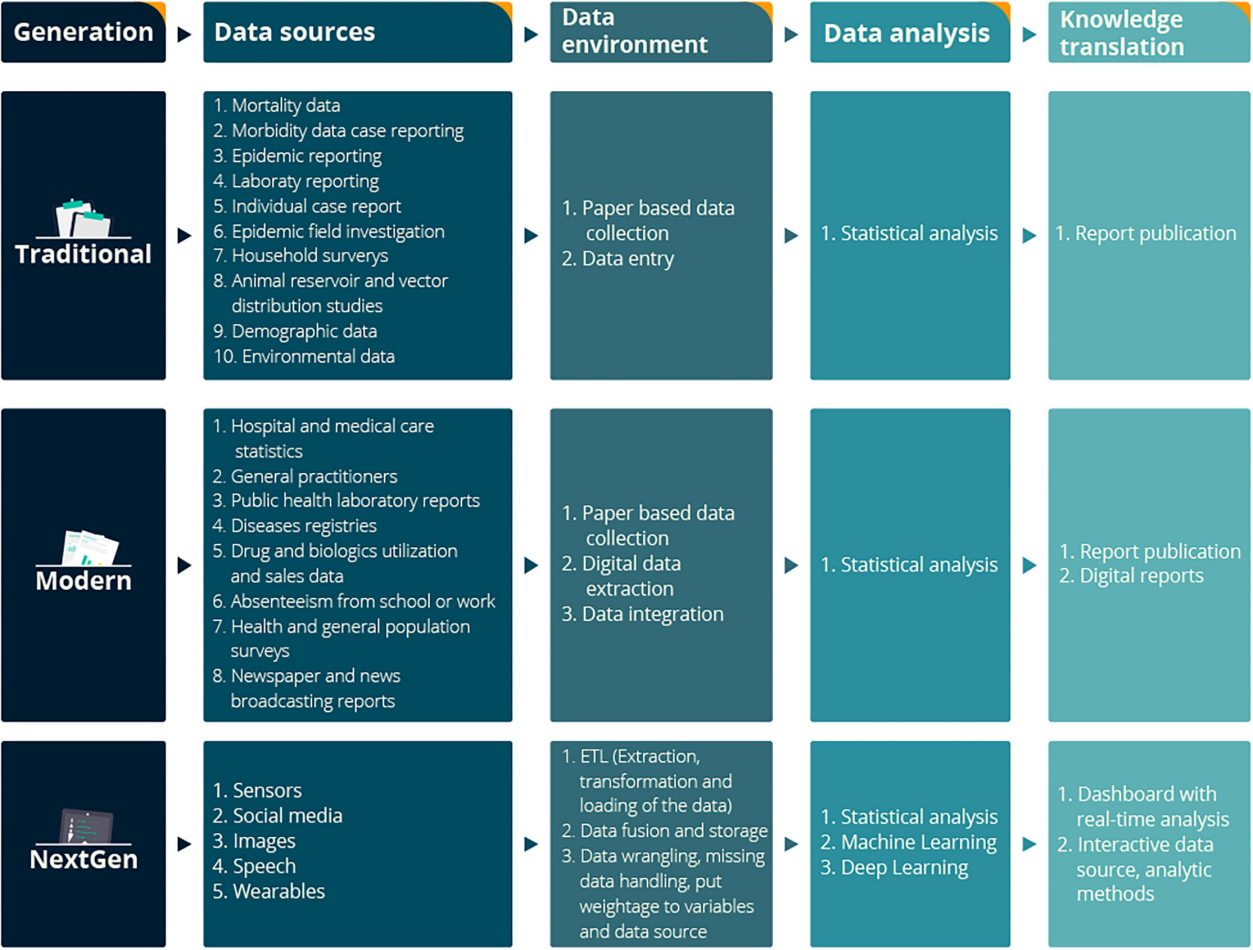
Conceptual framework of NextGen Public Health Surveillance with Traditional, Modern, and NextGen data sources. Traditional and modern data sources extracted from Declich and Carter (17).

Public health recommendations focus on the social determinants of health and health equity (16). Surveillance is the process by which ongoing health data are collected, analyzed, and reported, and it is critical to informing public health services. In 1968, the World Health Organization listed 10 essential data sources for surveillance (17) (Figure 1: Traditional data sources) that at the time relied on paper-based data collection and manual data entry. Surveillance capability has evolved enormously alongside advances in technology. It now includes digital data extracted from several sources (Figure 1: Modern data sources), offering reduced processing time, fewer errors, and reduced lag between data collection and its use.

The above said, surveillance data are still often obtained from questionnaire-based surveys online surveys, in-person or telephone-based interviews (18), and such data collection requires enormous resources and funding (19, 20). Data quality can be compromised by declining response rates (18), recall bias (21), and low granularity of the data (22) as in the traditional data collection system, there is a limited number of subjects provide their inputs. Without complete and comprehensive information, the value of the data reduced. For example, fewer subjects with a smaller *n*, really only impacts the precision of the estimates that come from surveillance. To further explain, the system might not get very precise incidence estimates, which may or may not be a problem depending on the goal of the system. The bigger issue with declining response rates is that they usually do not happen at random and meaning you're a less representative set of results. This is an issue if the factors that lead to making it into surveillance also relate to the issue you are trying to measure with the surveillance system. Current data used for the surveillance have challenges like missing data, under-reporting, inconsistencies, invalid data, illegible handwriting, non-standardization of vocabulary, measurement error, and inappropriate fields (23). Traditional data sources used in surveillance are often delayed. For example, at least 1 year is required for getting a Canadian Community Health Survey (CCHS) update. “Public Health Ontario” in Canada affirms interdependent gaps within surveillance, insufficient data to build comprehensive health indicators (24), and an absence of existing mechanisms to capture some of healthcare's vital components.

Current surveillance relies on both prospective and retrospective data collection, analysis, and reporting (25). The current pandemic has highlighted the essential need for real-time public health surveillance to improve the evidence-based decision-making process (26). Our evolving knowledge about chronic diseases, their risk factors, and management also demands the modernization of surveillance (25). Real-time responses to emerging public health threats require real-time and systematic data collection.

## Next-Generation Data Sources for Public Health Surveillance

Researchers have attempted to build and analyze health indicators using innovative data sources (27–29). They are exploring the use of smartphones (30), online searches (31), social media (7), wearables (32), ambient sensors (33), electronic health records (EHRs) (27, 34), medical-administrative records (27), and pharmacy sales (28) to broaden the scope of surveillance.

As a source of surveillance data, information technologies are potentially advantageous because their near-universal uptake by a significant portion of the population creates vast quantities and varieties of data (22). For example, wearable data from six billion nights has been used to understand sleep duration, quality, and change in pattern with time (35, 36). Effective use of big data for surveillance requires innovative analytical methods such as data integration (32) and data visualization (28, 37, 38). Big data analytics is becoming mainstream in public health, integrating knowledge and skills from health informatics and biostatistics (39).

## The Internet of Things as a Novel Data Source

The Internet of Things (IoT) is a technological innovation through which devices can communicate with each other in real-time through an internet connection (40). For example, several household devices are interconnected to achieve a common objective, such as monitoring temperature or motion (40). Integrated devices can include different sensors, mobile phones, mobile applications, wearable devices, and Radio-Frequency Identification (RFID) tags (40).

IoT devices have accelerated data collection (13, 41). Connectivity among people, machines, and organizations increases as device availability and affordability improve (22). This increase in connectivity is because of the ease of use of the devices, user-friendly designs, and internet speed. These parameters reduced the time gap within communication, broaden the scope of communication by providing different choice, be it audio visual, text, or hybrid of multiple methods. People can interact with the machines and vice versa, which was not possible earlier due to lack of technological progress. In 2011, the number of interconnected devices overtook the actual number of people globally (42). The potential for data generation is exponential (41). As the IoT data has already been successfully used in multiple setups to monitor individual health outcomes and report on environmental conditions, some of the best use cases has been described below.

### Use of IoT Data to Support Individual Health Outcomes

The management of chronic conditions has traditionally relied on patients interacting with their healthcare providers in person. However, patients spend most of their time outside the clinic. IoT monitoring provides an opportunity to collect real-time health information between patient-healthcare provider interactions.

Smart devices, such as wristbands, with IoT technology have been developed to measure individual physiological data, including physical activity (10, 43), sedentary time (44), oxygen saturation (45–47), heart rhythm (45, 46), muscle tremors (48), spinal posture (49), brainwaves (50), sleep (51), diet (52, 53), electrodermal activity monitoring for sympathetic response (44) and oral health care (54). With regards to specialized medical care, IoT technology has been used to cater to the need of cardiovascular (18), cardiopulmonary (18) and ophthalmology (55). With regards to different categories of populations, IoT has been used to help to monitor indicators related to women's health (56), including pregnancy (57), soldiers at the country borders (58), nursing care at the hospitals (59), the elderly population in the long-term-care homes (60), persons with neurological conditions at the rehabilitation center (49), and also for persons with respiratory complaints including asthma (61).

IoT devices have a multipurpose use within the healthcare field, such as their capabilities can range from providing prenatal care to rehabilitation to monitoring seniors or athletes. IoT devices have successfully provided real-time health information on maternal and fetal health between regular appointments (57). By monitoring vital signs using sensors, IoT platforms have been designed to provide people with diabetes with feedback and notifications to mitigate the risk of complications (62–64). Additionally, wearable devices have been used to detect falls and changes in behavioral activity for seniors living independently (65–68). Monitoring systems have also been developed to evaluate sports rehabilitation (69–72). IoT can support individual outcomes by allowing patients to manage their health outside of the clinical setting.

### Use of IoT Data to Monitor Environmental Conditions

The IoT can also monitor environmental conditions in areas where we live, work, and play. Monitoring air purification in hospital settings plays a role in mitigating hospital-related infections (73). Monitoring air quality is already used to quantify climate change impact (74) and has the potential to help mitigate its impact in the future (75). IoT has been employed to monitor hospital circulating air volume, ozone concentration, temperature, humidity, and leaked ultraviolet intensity (73). Preventive behavior like hand washing can also be monitored (76). Indicators of healthy outdoor environments, such as water pollution and air quality, have been another target of IoT health research (61, 77, 78).

## The Internet of Things in Public Health Surveillance

IoT data has been successfully used in other health domains but has not yet been fully used in public health. In response to the pandemic, the 2020 Riyadh Declaration made several recommendations to address the shortcomings in global public health response systems (79). The Declaration prioritized the need for scalable and sustainable digital health technologies and the adoption of health intelligence (79). There is a growing interest in using IoT data for building public health indicators at various levels (80–82).

### Advantages of IoT in Public Health Surveillance

IoT data have the potential to overcome shortcomings of current surveillance. IoT data sources provide high-frequency data with greater usability, and much of the device infrastructure for surveillance is already in place (i.e., smartphones, wearable technologies, internet access). Currently, worldwide more than three billion smartphone users (83), 722 million users of several kinds of wearable devices (84), and more than 1.2 billion smart-home connected devices exist (85). IoT data benefits from essential features like high granularity (22), objectivity (32), and validity (86). These “user-generated data ecosystems” are being generated with minimal effort by the device users and researchers. To date, the monetary cost to participants and researchers is low, suggesting that public health monitoring costs would likewise be minimal (87, 88). Finally, IoT enables near real-time data collection (89). This can significantly reduce the time gap between health events, data collection, reporting, and intervention.

Here we have assessed IoT's current attributes using the framework for evaluating public health surveillance by Groseclose et al. (90), which outlines nine features of surveillance systems to consider (Table 1). As summarized in the table, the major advantages of IoT data sources appear to be high-frequency data collection, the potential to have syndromic surveillance, zero effort data collection method, high volume, and variety of data. The major disadvantages appear to be lack of representativeness within a single data source, private players' involvement as the data owner, the need for a high technological system to store, clean, and analyze the data, and interoperability. In addition to the above points, data privacy concerns of users are a potential disadvantage of acceptance of this technology from the user point of view (81).

**Table 1:** Analysis of IoT as a data source for public health surveillance, using Groseclose et al. (90)^#^ framework for evaluating public health surveillance.

**Attributes (definition)**	**Features of IoT data**
**Simplicity** “*The system's structure and ease of operation. The system should be as simple as possible.”* (90)	• Data collection/extraction from users without complex interactions using Application Programming Interface (APIs) that the manufacturer often provides. • Easy access to the data, which is often collected by passive sensors, minimizing the burden for the user. • IoT systems rapidly generate large volumes of data in real-time, creating challenges associated with managing, hosting, and analyzing big data. • Diverse types of data being generated: numeric, images, text, or audio. • Collects vast amounts of data from the same individual, often supporting longitudinal analysis.
**Flexibility** “*Ability to adapt to changing information needs or technological operating conditions with little additional time, personnel, or allocated funds.”* (90)	• Application Programming Interfaces (APIs) make it easy to adapt to the technology to the end-users being used, type of data, type of database, type of storage, and security requirements. • New IoT data sources that use APIs can easily be integrated into systems, also affording changes in a data structure as technologies evolve. • Changes in case definition can be updated in algorithms rather than requiring changes to data collected since systems can access the raw data. • The system can be automated to generate alert systems without manual effort which can help public health officials identify potential signals for future outbreaks early.
**Data quality** “*Completeness and validity of the data recorded in the system.”* (90)	• IoT data often suffers from missing, inaccurate, and incomplete data. • Wearable sensors that require participants to recharge and remember to interact with the device often have larger volumes of missing data. • Ambient sensors often generate continuous and complete datasets as they are always connected, powered on, and streaming. • Technology development is leading to improved data quality across all IoT sensors.
**Acceptability** *"Willingness of persons and organizations to participate in the system.”* (90)	• IoT technologies are pervasive, and in the community, a part of the population is already using those technologies to generate data. • IoT adoption is accelerating in the last decade and is predicted to be much higher in the near future. • Recent advancement in technology used “skin interfaced sensors” not only monitor physical activities and vital signs but also keep track of molecular biomarkers of the human body (104). • Users need to agree to share their data, as it has already been collected.
**Sensitivity** “*At the level of case reporting: the proportion of cases of a disease or event detected by the system. Ability to detect outbreaks over time and evaluation of surveillance system.”* (90)	• IoT sensors, in most cases, do not focus on the detection of specific diseases such as COVID-19 or influenza but rather on symptoms like fever, abnormal heart rate, or change in gait pattern. • IoT technology is ideal for supporting syndromic surveillance by collecting data about healthy behaviors and health variables in real-time. • IoT technology will collect data that is often indirectly associated with health and health risk behaviors (e.g., indoor motion data to quantify sleep patterns, phone mobility data used to quantify response to COVID-10 policies). • IoT will provide extensive participant data with a higher likelihood of the presence of events. • The longitudinal nature of the data can detect future anomalies using Artificial Intelligence models within the healthcare sector and send alerts to policymakers. The longitudinal and continuity nature of the data will provide richer insights into population behaviors, which increases the likelihood of getting the events of interest.
**Positive predictive value** “*The proportion of reported cases that actually have the event under surveillance.”* (90)	• The proportion of the presence of IoT within the community is increasing and predicting the true positive cases will be easier using IoT data by identifying early alerts. • The detection of specific diseases is possible, as technologies such as lab on a chip (106, 107) allow for real-time detection of pathogens and contaminants. • Positive predictive value seems to be in a disadvantageous position with the current IoT data environment, but this might change in the future.
**Representativeness** “*Ability to accurately describe the occurrence of a health-related event overtime and distribution of the population by place and person.”* (90)	• Large number of participants can provide access to data who were not represented in the traditional data collection method. • IoT technologies are ubiquitous, highly pervasive, and are generating data 24/7. • Data mining from sensors already owned by the population generates a biased sample, with data from the wealthier and more physically active part of the population. • Studies can supplement biased samples by deploying targeted studies to collect data from under-represented subgroups of the population.
**Timeliness** “*Reflects the speed between steps in a system.”* (90)	• Data is often collected at high frequencies, often affording access to data in the near real-time. • An increase in the data's granularity and the longitudinal nature of the data can provide richer insights, for instance, faster alerts of anomalies for specific health issues, and support the creation of innovative indicators. • In the near future, the IoT data source may become helpful to identify future pandemic and climate-related emergencies. Immediate assessment of the impact of policy changes (for example, “work from home” during the pandemic) can be possible using IoT data. • Improvement from traditional data sources where data collection often happens once yearly or less frequently.
**Stability** “*Ability to rely on the system for availability and to collect manage and provide data without failure. Ability to be operational when needed.”* (90)	• Private cloud systems can provide the necessary data security and maintain the users' privacy. • Redundant, always available, more stable public health surveillance platforms/systems can be built using private cloud solutions, having the capacity to collect uninterrupted data without failure. IoT manufacturers and IoT data custodians can deliver such redundant and stable systems for their consumers' everyday use. • The disadvantage of these IoT data manufacturers is ever-changing company environment (for example, corporate and big private entities) might not provide a stable source of data. The alternative source of data should be listed as a backup plan to support and strengthen when required.

# *Groseclose et al. (90)*.

### Challenges to Using IoT in Public Health Surveillance

The challenge now is how to access and analyze the data being gathered. Some IoT companies create sharable, research-oriented data sources, such as “donate your data” from ecobee, a smart thermostat company in Canada (91). ecobee's smart home products include motion and temperature sensors, and research teams have access to longitudinal data from thousands of households with a data granularity of 5 min intervals.

Other IoT companies publish studies from their own smart devices using artificial intelligence algorithms for population-level measurements. For example, Fitbit wearables recorded sleep data from over six billion nights of its customers' sleep (35), the most prominent sleep dataset ever collected. Similarly, Oura Health used IoT data gathered from their Oura ring, a wearable sensor that tracks key signals from the human body (sleep, heart rate, skin temperature, physical activity), delivering critical insights to help an individual harness their body's potential daily and also to monitor vital health indicators (92).

Another hurdle is the ability to fuse data from multiple devices to produce a unified result. Several research projects have focused on making IoT data fusion viable in the real world by designing computing infrastructure and data fusion techniques (89, 93). Real-time IoT analysis from multiple health monitoring devices may overwhelm current computational capabilities, such as using multiple devices to monitor each football player's physiological indicators during a game (94). A distributed computational framework to handle complex computational needs was developed by Higinio et al. for health surveillance (94). The use of each smart devices' computing capabilities effectively shared advanced health monitoring applications (94).

Regarding technical challenges related to IoT, some of the critical issues are energy optimization, hardware compatibility, security, and data connectivity (95). A recent study by Iwendi et al. in 2020 shows that there are certain highly specialized algorithm such as a “hybrid meta-heuristic algorithm” has the potential to optimize the energy consumption of the sensors related to wireless sensor networks (95).

Aberration detection identifies unusual incidents or information trends with possible significance to clinical or public health (96). Methods for detecting such aberrations have also evolved significantly. Current modeling methods can now analyze individual surveillance data collected from different sources and integrate multiple covariates (97). The algorithms used for signal recognition have improved over the last decade and are now better equipped to utilize advanced informatics to capture surveillance data aberrations (96, 97) accurately.

In 2018, Faverjon C. and Berezowski J. elaborated on IoT data's utility for aberration detection (97, 98). Two studies have shown that user data from wearables (Fitbit and the Oura ring) could detect early signs of COVID-19 infection (99–103). Evidence shows the risk of hospitalization related to COVID-19 can be calculated from self-reported symptoms and predictive physiological signs by combining different health and behavioral data from consumer wearable devices; this may help identify pathological changes weeks before observation using traditional epidemiological monitoring (99, 100). As described in the study using Fitbit wearable, it has the potential to detect almost half of COVID-19 positive cases 24 h before participants reported the onset of symptoms with 70 percent specificity (103). Besides joint effort by multiple countries to develop vaccines and potential drugs to prevent and treat COVID-19, skin-integrated and skin interfaced sensors, positioned at optimal locations of the body, might address the ongoing and critical need for objective, continuous, and sensitive tools to detect COVID-19 symptoms early in the general population (101, 104). A research study highlighted a practical approach for managing epidemics using digital technologies with a roadmap to a rapid and universal diagnostic method for the population level detection of several respiratory infections in advance of symptoms (102). These anomalies could predict future outbreaks (97) and prevent the spread of infectious diseases (105).

## Nextgen Public Health Surveillance

The COVID-19 pandemic has revealed a need to strengthen our public health surveillance and response systems. With the availability of public data and advances in collection and analysis, there is an opportunity to strengthen existing surveillance systems by harnessing complementary data sources like IoT-based data (31).

Figure 1 describes the NextGen surveillance systems' conceptual framework. The first layer describes the sources of public health data. The second layer represents the data architecture. Once the data integration process is completed, data manipulation and analysis can be possible using statistics, machine learning, and deep learning algorithms. This process will help discover new public health indicators and advance our understanding of existing disease risk factors.

## Conclusion

Current public health surveillance systems have unique challenges in getting the relevant data at the right time and utilizing those data sources for policy-level decision-making. There is a considerable volume of non-traditional data being self-generated by the public through their ubiquitous use of smart devices. Public health has the potential to utilize the real-time, longitudinal data collected through the Internet of Things (IoT) necessary for health surveillance. Advances in computing are now bringing IoT-based surveillance into the realm of possibility. The advantages of IoT data include high-frequency, high volume, zero effort data collection method, with a potential to have syndromic surveillance.

## Data Availability Statement

The original contributions presented in the study are included in the article/supplementary material, further inquiries can be directed to the corresponding author.

## Author Contributions

KS and PM developed the theoretical framework for the paper. PM supervised KS for the project. KS wrote the first draft of the manuscript with input from all authors. SM and JD helped provide overall direction and planning. All authors contributed to manuscript reading and revision and have approved the submitted version.

## Funding

This work was supported by Natural Sciences and Engineering Research Council of Canada (RPGIN-2017-05310) and the Ontario Centre of Innovation (33080).

## Conflict of Interest

The authors declare that the research was conducted in the absence of any commercial or financial relationships that could be construed as a potential conflict of interest.

## Publisher's Note

All claims expressed in this article are solely those of the authors and do not necessarily represent those of their affiliated organizations, or those of the publisher, the editors and the reviewers. Any product that may be evaluated in this article, or claim that may be made by its manufacturer, is not guaranteed or endorsed by the publisher.
